# Quantifying the uncertainty of LLM hallucination spreading in complex adaptive social networks

**DOI:** 10.1038/s41598-024-66708-4

**Published:** 2024-07-16

**Authors:** Guozhi Hao, Jun Wu, Qianqian Pan, Rosario Morello

**Affiliations:** 1 Graduate School of Information, Production and Systems, Waseda University, Fukuoka, 808-0135 Japan; 2 Department of Information Engineering, Infrastructure and Sustainable Energy, University Mediterranea of Reggio Calabria, Via Graziella, Reggio Calabria, 89122 Italy

**Keywords:** Computer science, Information technology

## Abstract

Large language models (LLMs) are becoming a significant source of content generation in social networks, which is a typical complex adaptive system (CAS). However, due to their hallucinatory nature, LLMs produce false information that can spread through social networks, which will impact the stability of the whole society. The uncertainty of LLMs false information spread within social networks is attributable to the diversity of individual behaviors, intricate interconnectivity, and dynamic network structures. Quantifying the uncertainty of false information spread by LLMs in social networks is beneficial for preemptively devising strategies to defend against threats. To address these challenges, we propose an LLMs hallucination-aware dynamic modeling method via agent-based probability distributions, spread popularity and community affiliation, to quantify the uncertain spreading of LLMs hallucination in social networks. We set up the node attributes and behaviors in the model based on real-world data. For evaluation, we consider the spreaders, informed people, discerning and unwilling non-spreaders as indicators, and quantified the spreading under different LLMs task situations, such as QA, dialogue, and summarization, as well as LLMs versions. Furthermore, we conduct experiments using real-world LLM hallucination data combined with social network features to ensure the validity of the proposed quantifying scheme.

## Introduction

Large language models (LLMs) have become increasingly mainstream AI technology for kinds of intelligent applications since the publishing of OpenAI’s ChatGenerative Pre-trained Transformer (ChatGPT)^1^. The following LLMs are also popular today like Meta’s LLaMA^2^, Google’s Bard^3^. These models have revolutionized content creation through their ability to process and generate text based on immense datasets. They use advanced algorithms to learn patterns and structures in language, enabling them to produce coherent and contextually relevant text. LLMs’ content generation capabilities will power various domains, bringing smarter, automated functionality^4^. In the medical field, the content generated by LLMs will assist physicians in diagnostic treatments^5,6^. LLMs can also generate code^7,8^, optimize translation results^9,10^. While LLMs bring many enhancements, they also bring some security concerns. LLMs may produce information that appears to be true but is false and useless, a phenomenon known as hallucination. The hallucinatory information may be contrary to the underlying facts or irrelevant to the user-entered prompt^11,12^. The phenomenon of hallucinations in LLMs from their training process, has been widely observed in current mainstream LLMs. LLMs learn a wide range of knowledge during the pre-training process. In the fine-tuning process, manual labeling gives the big model the ability to imitate real answers. If a question involves untrained knowledge, LLMs don’t simply say they don’t know. Instead, they generate plausible but potentially inaccurate responses^13^.

**Figure 1:**
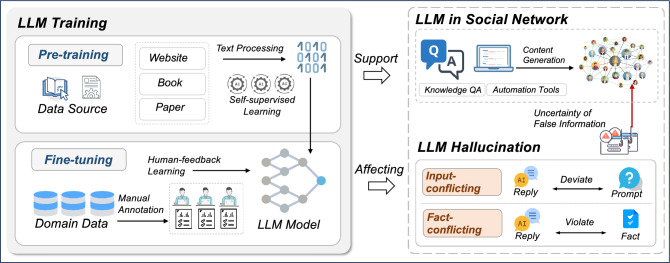
The scenario of LLMs-generated hallucinatory false information spreading in social networks.

The security of social networks has been studied extensively^14,15^, but LLMs present new challenges. Currently, LLMs such as ChatGPT have been widely used for knowledge question and answer (QA), image generation, article writing, code generation, and other applications. In these processes, the hallucinatory false information that may be generated by LLMs will be spread through social networks as shown in Fig. 1. This phenomenon not only distorts the information ecosystem but also potentially influences public opinion and decision-making processes based on inaccuracies. The nature of social networks to quickly spread content makes this problem worse. It highlights the urgent need to measure and assess how AI creates and shares content. As a complex adaptive system, quantitatively analyzing the phenomena in social networks will help us understand the functioning of social networks and address the challenges of the new risk of spreading false information posed by LLMs hallucination^16,17^. Currently, traditional complex adaptive system (CAS) based approaches to modeling social networks typically address socio-economics-related problems, such as supply chain^18^ and community structure^19^. Therefore, confronting the new problems posed by LLMs, there is a necessity to design a new social network modeling approach specific to the hallucinatory false information of LLMs, for quantitative evaluation of spreading.

To address the challenges mentioned above, we design a new agent-based modeling (ABM) approach for social networks. In CAS, agents often represent individuals or entities in a network, each following simple rules. Through ABM, researchers can observe emergent phenomena and complex dynamics arising from the collective behavior of agents. This approach is particularly valuable in fields like ecology^20^, economics^21^, and social sciences^22^, where understanding the interplay of individual actions and systemic patterns is crucial. The model’s strength lies in its ability to capture non-linearities and feedback loops, providing insights into the adaptive and evolving nature of complex systems. In our ABM approach, we define the properties of hallucinatory false information of LLMs spreading in social networks and obtain the characteristics of hallucinatory false information under different task scenarios, and different model versions by testing the current mainstream LLMs. We designed the attributes of agent in the model based on real-world data and real Facebook social network, in which the discernment ability of user agent is based on its education level, and the ability to recognise false information is based on out-degree centrality in the social network. User agent’s spreading behaviour towards false information is based on behavioural science, we considered novelty preference, domain expertise effect and group polarization to design agent behaviour. Finally, the model allows us to quantitatively analyze the spreading of LLMs hallucinatory false information under different situations in social networks, we set up different application scenarios and versions of LLMs to count the changes of different characters in the process of spreading false information, including spreaders, discerning non-spreaders, unwilling non-spreaders and informed people.

## Methods

**Figure 2:**
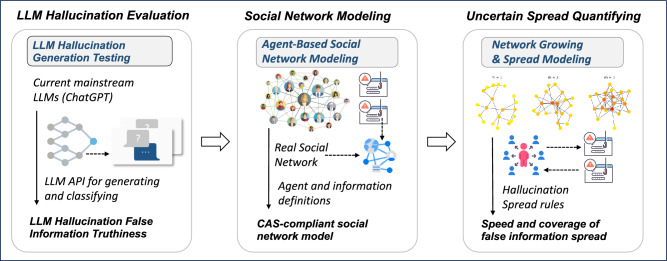
Architecture of our proposed quantitative approach to social networks.

To quantify the uncertainty of LLM-generated hallucinatory false information in social networks. Our proposed quantitative approach consists of three components, including LLM hallucination evaluation, social network modeling, and uncertain spread quantifying as shown in Fig. 2. First, we checked the truthfulness of false information from LLMs under various conditions by changing the model versions, temperature settings, and LLM tasks like question answering, conversation, and summarization. We also tested different generation methods, such as single turn, multiple turns, and filtering. Second, we modeled social networks using the agent-based modeling approach based on real Facebook social network data. The model includes attributes of social network agents, attributes of LLM hallucination information, and the network construction. ABM allowed us to simulate and analyze the dynamics within the network. By incorporating the real social structure and data, the model aimed to provide insights into the potential spread and impact of misinformation or distorted information generated by LLMs within social networks. Third, we considered the spreading rules of LLM hallucination from a behavioral science perspective, based on real-world social network users’ behavioral habits. We took into account factors such as the popularity of the message, cognitive biases of users, and the familiarity of the message to understand their impact on spreading.

### LLM hallucination evaluation

**Figure 3:**
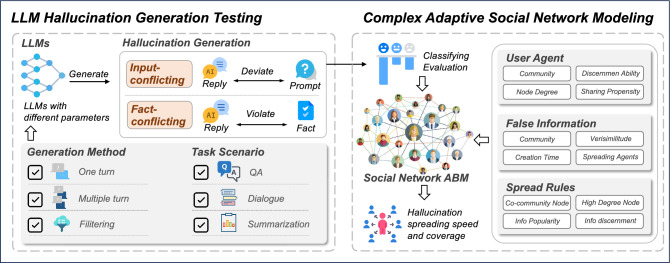
Technical details of our proposed quantitative approach to social networks.

The hallucinatory nature of LLM will produce information that appears real but is actually false. We tested and evaluated the hallucinatory false information generated by the LLM in the method shown in Fig. 3. We implement the hallucination evaluation benchmark HaluEval to generate the LLM hallucination content^23^. The data for the knowledge QA comes from the database HotpotQA^24^. In task QA, LLM will give the user knowledge question answers based on the knowledge from HotpotQA, such as “It is a hygroscopic solid that is highly soluble in water and slightly soluble in alcohol. Ethanol, also called alcohol, ethyl alcohol, and drinking alcohol, is a compound and simple alcohol with the chemical formula C2H5OH...”, the user can ask the question “Cadmium Chloride is slightly soluble in this chemical, it is also called what?” and the LLM need to give the answer. The data for the dialogue comes from the database OpenDiaKG^25^. In the task dialogue, LLM chats with a user based on knowledge, such as “Floyd Mayweather, Jr. is a/an Boxer...”, user can give some questions, like “Do you know who Floyd Mayweather, Jr is?”, then LLM replies “ Do you mean Money Mayweather?”, user said “Is that a nickname for Floyd?”, LLM reply “yes, do you know why?”, user said “ I have no idea. I don’t know who he is”, and then LLM should explain their early reply. The data for the summarization comes from the database CNN-Dailymail^26^. In this task scenario, the user will give the LLM a paragraph, which the LLM needs to summarize. Based on the above three task scenarios, we obtained the hallucinatory false information of LLM through three generation methods. The first method is one turn, which we directly feed the instruction into the LLM model and generate a hallucinated answer. Second, we give the hallucination complete instruction using multiple turn conversations to make sure the LLM model knows how to generate hallucinations. Third, we use the instruction of hallucination filtering to select the better hallucination generation from one turn and multiple turn methods.

In our experiments, we observed two kinds of hallucinatory contents, input-conflicting generation and fact-conflicting generation. On the one hand, input-conflicting hallucination deviates from the input prompt of the users. For example, based on the knowledge “Robert Lewis Glenister (born 11th March 1960) is an English actor known for his roles as con man Ash ’Three Socks’ Morgan in the BBC television series “Hustle” and Nicholas Blake in the BBC spy drama ’Spooks’...”, the users give the question “Close to the Enemy starred the English actor known as Ash Morgan in what BBC series?”, the right answer is “Hustle”, but LLM gives the hallucination answer “Close to the Enemy starred the actor Nicholas Blake in the BBC spy drama.” Hallucinatory false content does not answer the user’s question, in some automated LLM-based QA systems, users may release this false information in social networks without reviewing it. On the other hand, fact-conflicting hallucination is not faithful to established world knowledge. In our previous dialogue example, the right response should be “He is a boxer, and he is the highest paid athlete on this rock we call home.”, the hallucination response is “He is a singer, and he is the highest paid musician on this rock we call home.”. After producing false information, we use an LLM model to classify whether the information is indeed false. The accuracy of this classification shows how hard it is to identify the false information. The difficulty of recognizing hallucinatory false information in a social network will affect the spreading in the social network.

### Social network modeling

We study social networks as a CAS, and we apply the ABM method in CAS to the modeling of social networks^27^. Figure 3 shows the details of our complex adaptive social network modeling. In the following sections, we describe the process of our social network modeling.

#### User agent in social network

In modeling the social network agent, we designed our ABM method based on the characteristics of the real Facebook social network^28^. We assigned attributes to agents in our model, including the sharing propensity that follows the distribution of out-degree centrality from the real network, the discernment based on the distribution of education levels in the real world, community attributes that can be adjusted according to the application scenario, and node degree. These attributes were designed to reflect how individuals in a social network might behave in terms of spreading information, evaluating the credibility of the information they encounter, and interacting within their social circles. The following content is detailed formal descriptions:

- Sharing propensity: In social network analysis, out-degree centrality measures a node’s ability to send connections (such as information, resources, or social interactions) to other nodes. A node with high out-degree centrality indicates a tendency to actively share information, resources, or establish connections with other nodes, thus can be considered as having a higher willingness to share. Observing the distribution of out-degree centrality in the real Facebook network, through experimentation and fitting, it was found that the frequency distribution of its values conforms to a power-law distribution minus a constant, with the frequency values having a minimum of 0. This indicates that in the Facebook social network, a small number of nodes have a very high out-degree centrality, meaning they share connections with many other nodes. So, we present the probability distribution of agent sharing propensity $S_i$ as shown in Eq. (1). $\alpha$, *C* and *k* are parameters of the probability distribution that can be determined through experimental fitting. Using our model, researchers can also set different parameters according to the objectives of their study to generate various node characteristics for simulating the spread of LLM hallucination information. For agent *i* in social network, *N* is the number of nodes, the sharing propensity $S_i$ is given by the Eq. (2).
  1 $$\begin{aligned}{} & {} p(S_i) = \max \left( 0, CS_i^{-\alpha } - k\right) \quad \text {for} \quad S_i \in [0, 1] \end{aligned}$$
  2 $$\begin{aligned}{} & {} S_i \sim p(S_i) \quad \text {for} \quad i = 1, \ldots , N \end{aligned}$$
- Discernment ability: In real social networks, because of the different education levels and knowledge reserves, different groups have different abilities to recognize false information. Based on experiments with real-world educational data, we found that the education level of individuals conforms to a truncated normal distribution, which means that a small number of people have a strong or weak ability to recognize false information and the majority of people’s ability to recognize false information lies between a mean value. Each agent’s ability to discern false information is modeled by a discernment ability $d_i$. The discernment ability reflects the ability of user *i* to differentiate between false information and genuine information, which can be expressed as shown in Eq. (3), *a* and *b* are the lower and upper limits of the truncation interval respectively, $\mu$ is the mean of the untruncated normal distribution and $\sigma$ is the standard deviation of the untruncated normal distribution.
  3 $$\begin{aligned} f(d_i) = \frac{\phi \left( \frac{d_i - \mu }{\sigma }\right) }{\sigma \left( \Phi \left( \frac{b - \mu }{\sigma }\right) - \Phi \left( \frac{a - \mu }{\sigma }\right) \right) } \quad \text {for} \quad a \le d_i \le b \end{aligned}$$
- Community affiliation: For real social networks, researchers can set different community attributes in our model based on their testing requirements. For example, when using our model to test the prevalence of LLM hallucination information in a campus setting, different grades can be set as community attributes along with corresponding other parameters to test the impact of specific groups on the spreading. Each user agent *i* is assigned to a single community $c_i$ where $c_i \in \mathbb {C}$, and $\mathbb {C} = \{1, 2, \dots , C\}$ represents the set of all communities within the network, with *C* being the total number of distinct communities. We argue that social affiliation, where users in the network have different communities, and community affiliation will affect users’ behavior in spreading false information.
- Node degree: The node degree $\kappa _i$ of an agent *i* quantifies the number of social ties that the agent has within the network graph. Formally, the network is represented as an undirected graph $G = (N, E)$ where *N* is the set of nodes corresponding to user agents and *E* is the set of edges representing the social ties. The degree $\kappa _i$ of agent *i* can be mathematically expressed as: $\kappa _i = |\{j \in N \; | \; (i, j) \in E\}|$.

Combining all the above elements, we can construct a model that captures the agents’ interconnectedness, their ability to act as conduits for the spread of false information, and the impact of their characteristics on this process. The behavior of each user agent and the dynamics of false information spread can be mathematically analyzed to understand the patterns of LLM false information in social networks.

#### Social network structure

We construct the network structure in our model based on the structural characteristics of real social networks. The Barabási–Albert (BA) model offers a robust framework for mirroring the inherent scale-free nature of real social networks, where a small subset of nodes holds a disproportionately large number of connections. We think this characteristic is pivotal, as it aligns closely with real-world social network dynamics, where key individuals can significantly impact information spreading and network structure. The simplicity and generality of the BA model, grounded in its core principles of growth and preferential attachment, facilitate a comprehensive analysis of network evolution over time. So, we apply BA model to construct our social network. For initiation, the social network is represented as a graph $G_t = (N_t, E_t)$ at time step *t*, where $N_t$ denotes the set of nodes (agents) and $E_t$ denotes the set of edges (social ties). At each subsequent time step $t+1$, the network grows according to the following procedure. First is node addition, a pre-defined number of new agents $\Delta n$ are added to the network. For each new agent *i*, we have $N_{t+1} = N_t \cup \{i\}$. Second is preferential attachment, every new agent *i* forms a single social tie with an existing agent $j \in N_t$ probability proportional to the degree $\kappa _j$ of agent *j*. That is, the probability $P(i \rightarrow j)$ of forming an edge between new agent *i* and existing agent *j* is given by:

4 $$\begin{aligned} P(i \rightarrow j) = \frac{\kappa _j}{\sum _{k \in N_t} \kappa _k} \end{aligned}$$

Then, the network graph is updated to $G_{t+1} = (N_{t+1}, E_{t+1})$ to reflect the new agents and edges. The whole process can be represented by the Algorithm 1.

**Algorithm 1:**
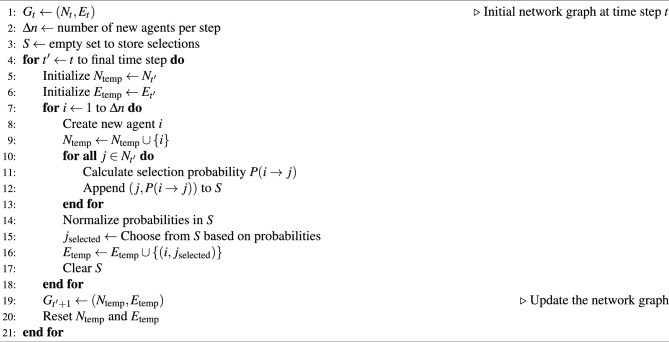
Social Network Growth via Barabási–Albert Model

#### False information in social network

In our model, the spread of false information generated by LLMs in social networks is influenced by several factors, including the information’s community affinity, its truthfulness, the time of creation, and the agents involved in its distribution. Here’s how we mathematically model these aspects of false information:

- Community affinity: We believe that false information should also have community, in which a certain group of people in a social network will be more familiar with a specific type of false information, which will influence users’ recognition of false information. The false information *f* is affiliated with a community $c_f \in \mathbb {C}$, where $\mathbb {C}$ denotes the set of all possible communities within the network, as previously defined. The community affinity $c_f$ implies that false information might have a higher likelihood of initial acceptance or spread within the community to which it is aligned.
- Truthfulness: The truthfulness of LLMs hallucinatory false information is related to our previous evaluation, when the accuracy of the hallucinatory false information being classified correctly is higher, it means that the false information is less truthful and is more likely to be recognized as false, when the accuracy of the hallucinatory false information being classified correctly is lower, then the truthfulness of the false information is higher, and the probability of the user to recognize the false information is lower. The truthfulness $V_f$ of false information *f* represents its perceived credibility to truth and takes on a value in the closed interval [0, 1] where 0 is completely implausible and 1 is indistinguishable from truth. This attribute influences the probability of acceptance and spread among user agents, where higher verisimilitude can lower the discernment efficacy.
- Creation time: The creation time $t_{\text {create}}^f$ of false information *f* denotes the time step during the simulation at which the false information originated. It allows for tracking the age of the false information as the simulation progresses.
- Spreading agents: The false information *f* maintains a set $A_f$ consisting of unique agent identifiers for those agents who have spread this false information. This set begins with the originator agent and expands as other agents share *f*. *N* represents the set of all agents within the network.
  5 $$\begin{aligned} A_f = \{i \in N \;|\; \text {agent }i\text { spreads false information }f\} \end{aligned}$$

Incorporating these attributes, the modeling of false information in a social network can be concisely expressed and analyzed, enabling an understanding of the dynamics involved in its spread relative to time, community structures, and the perceived credibility that it holds among users.

### Uncertain spreading quantifying

For quantifying the uncertain spreading of LLM hallucination in social networks, we integrated behavioral science and referred to the behavioral habits of users in real social networks to establish the propagation process. We considered four typical groups of people in the scenario of false information spreading as indicators, demonstrating the simulation results of our model, which will show as follows:

- Spreader (SP): Nodes in social networks that spread false information to their neighbours.
- Informed people (IP): Nodes in social networks that have received false information.
- Discerning non-spreaders (DN): Nodes in social networks that recognise false information and do not spread it.
- Unwilling non-spreaders (UN): Nodes in social networks that do not recognise false information and do not spread it.

The number of spreaders can illustrate the speed of false information spreading and observe changes in its propagation. The number of informed people can be used to assess the spread of false information throughout the social network. Discerning non-spreaders, as the most rational nodes in the network, play a role in preventing the spread of misinformation. Unwilling non-spreaders have not identified the false information but do not spread it due to a lack of willingness to do so. Based on user behavior in real social networks and behavioral science, we designed the rules for nodes to spread false information in our model. The detailed definitions are introduced as follows:

- Novelty preference: In social networks, a preference for novelty manifests as users seek to share novel information that has not been encountered before, because such content is more likely to attract attention, spark discussions, and repeated information may lead to a decline in the recipient’s attention or even cause annoyance. Therefore, in social networks, people are more inclined to spread trending information and are generally less willing to spread outdated messages. Additionally, individuals tend to avoid sharing the same information with the same contact repeatedly. Based on our definitions of spreading characters and behaviors, we have formalized the modeling of spreading parameters and processes. The popularity $P_f(t)$ of false information *f* at a given time step *t* is calculated as the ratio of the number of spreading agents to the duration for which the false information has been in the system:
  6 $$\begin{aligned} P_f(t) = \frac{|A_f|}{t - t_{\text {create}}^f}, \quad \text {for } t > t_{\text {create}}^f \end{aligned}$$
  where $|A_f|$ denotes the cardinality of set $A_f$, i.e., the total number of spreading agents, and the denominator is the time elapsed since the creation of *f*.
- Domain expertise effect: In the context of social networks, when people encounter information within their areas of expertise, their knowledge enables them to more easily discern the truthfulness and significance of the information, as well as to more effectively learn from and absorb knowledge.
- Group polarization: People tend to filter and interpret information based on their social identity and group affiliations. If a source of information is perceived as part of “our group,” then information from that source is more likely to be accepted. This sense of group identity can amplify the spread of misinformation. Based on the settings of our node spreading behaviour, the discernment ability of the node $d_i^t$ will be adjusted, when the node *i* belongs to the same community as the false information *f*, i.e., $c_i = c_f$, the node will enhance the discernment ability of the false information $\Delta _{\text {fcomm}}$ every time cycle because of domain expertise effect. The $I_{\{\cdot \}}$ denotes the indicator function if its condition is true and the constants $\Delta _{\text {fcomm}}$ and $\Delta _{\text {acomm}}$ represent the respective changes in discernment ability. When a node *i* receives false information *f* from a node $i'$ of the same community, i.e., $c_i = c_{i'}$, its recognition ability is adjusted $\Delta _{\text {acomm}}$ because of group polarization:
  7 $$\begin{aligned} d_i^t = d_i^{t-1} + I(c_i = c_f) \Delta _{\text {fcomm}} + I(c_i = c_{i'}) \Delta _{\text {acomm}} \end{aligned}$$
- Recognition of false information and the willingness in sharing: This is the foundational rule of our spreading model, where we have defined node attributes based on the characteristics of real social networks. Users will discern spreading based on their level of education. Nodes that are unable to identify the false information will then proceed to a dissemination decision, with the willingness to spread, serving as the probability of spreading.The effective discernment $d_i^t$ is compared to the information truthfulness $V_f$:
  8 $$\begin{aligned} \text {Spread}_i^f = {\left\{ \begin{array}{ll} 1, &{} \text {if } d_i^t \le V_f, \\ 0, &{} \text {otherwise}. \end{array}\right. } \end{aligned}$$
  Where $\text {Spread}_i^f$ indicates whether agent *i* decide to spread *f*. If the discernment ability is less than the truthfulness of the information, the node did not recognise the false information, and conversely, it did. Now that we have completed the node’s judgement of the false information, the node’s next spreading behaviour will depend on its spreading willingness $S_i^{t}$. Because of novelty preference, the node’s willingness to spread will receive the influence of information popularity $P_f(t)$, the *N* represents the number of nodes in network. We adjusted the node’s willingness to share:
  9 $$\begin{aligned} S_i^{t} = \max \left( 0, \min \left( S_i^{(t-1)} + \frac{P_f(t)}{N}, 1\right) \right) \end{aligned}$$

We describe the entire process of user agents spreading false information as an Algorithm 2. Based on the network structure obtained in Algorithm 1, we first initialise the node attributes and false information attributes in the social network. We randomly choose a community attribute of nodes as the initial node for false information infection, false information and nodes have the same community attribute, which reflects the fact that in real life people spend most of their time browsing information related to them. Next we iterate the spreading, adjusting and updating the node’s discernment ability, willingness to share and the popularity of the information according to the previous formula, and the truthfulness of the information and other attributes for the spreading judgement. Based on the spreading results we counted the number of different spreading characters. In this algorithm, rand(0, 1) generates a uniform random number in the interval [0, 1]. The algorithm aims to simulate the spread of information *f* in social network, based on users discernment ability, community affinity, sharing propensity, the truthfulness and community affinity of false information. We implement in our algorithm the effects of the behavioural sciences we mentioned on the spread of false information, including the effect of information popularity on willingness to spread due to novelty preference, and the effect of domain expertise effect and group polarization on users’ ability to discern false information.

**Algorithm 2:**
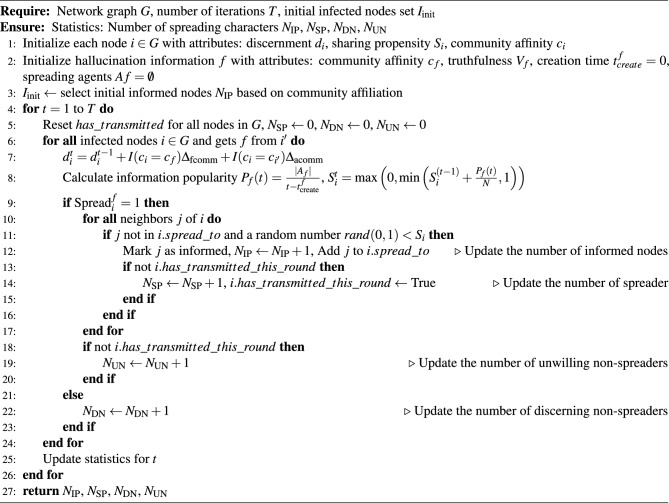
False Information Spreading Model

## Results

In the experimental part, we first evaluated the truthfulness of the hallucinatory false information generated by the current LLMs, considering different model versions, generation methods, application scenarios, and model temperature parameters. Next we demonstrate the ability of our model to characterize the spreading of false information in the real world in terms of parameter settings as well as spreading results. Finally, we evaluated the spreading of LLM-generated hallucinatory false information in social networks based on our model. The experiment is deployed on a server with Ubuntu 20.04LTS, CPU Xeon Gold 6326 2.9 GHz, 256G DDR4 RAM, 8TB SSD, and GPU NVIDIA RTX A5500. We give details of the experimental results in the following sections.

### LLM hallucination evaluation

**Figure 4:**
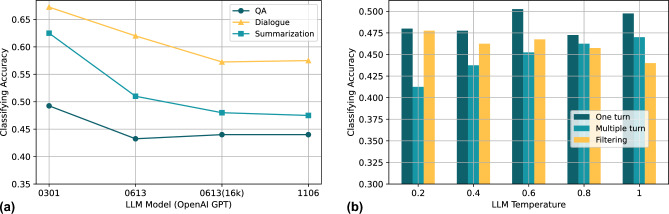
LLM hallucination false information evaluation. (**a**) Different LLM model versions and task scenarios (**b**) Different model temperature and methods of generation.

As shown in Fig. 4, we test the LLM hallucination generation under different situations, each test made at least 500 LLMs API requests. OpenAI GPT-3.5 is currently the mainstream and stabilized LLM, and we have tested four different versions of it by using its API, including GPT-3.5-turbo-0301, GPT-3.5-turbo-0613, GPT-3.5-turbo-16k-0613, GPT-3.5-turbo-1103. These models are released at different times, where the number at the end of the name represents its release date, and the newly released models usually have stronger generation capabilities. We used GPT-3.5-turbo-1103 to classify whether or not it was LLM hallucinatory false information. As shown in Fig. 4a, in the three task scenarios, the GPT-3.5-turbo-1103-generated hallucinatory false information has the lowest classifying accuracy, indicating that it is difficult to recognize it as false information, and based on the experimental results we can also assume that the hallucinatory false information generated by the new version of the model is less likely to be recognized. Besides, hallucinatory false information generated in the QA task was the most difficult to identify, followed by summarization, and hallucinatory false information generated in the dialogue task was the easiest to identify. Furthermore, as shown in Fig. 4b, we tested the hallucinatory false information generated by the GPT-3.5-turbo-1103 through different methods with different temperature parameters. Under the one turn approach, classification accuracy did not show a correlation with temperature, under the multiple turn approach, model-generated hallucinatory false information will be more easily identified as temperature increases, and under the filtering approach, model-generated hallucinatory false information will be more difficult to identify as temperature increases.

### Ability of the model to explain the real world

**Figure 5:**
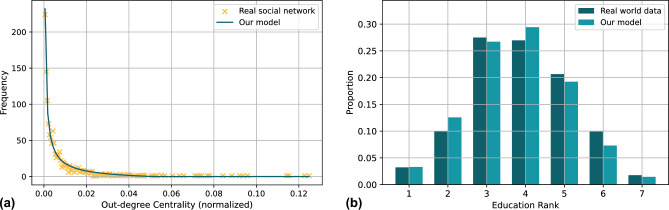
Ability of model parameters to simulate real-world data. (**a**) Simulated and real values of node out-degree centrality. (**b**) Simulated and real values of education rank.

To critically evaluate how our assumptions might influence the model’s ability to represent real-world phenomena accurately. We evaluated the attribute settings of the model nodes based on the characteristics of real social networks as well as real-world data, in addition, the hallucination generated by LLM can be viewed as a special kind of rumor, and we compared the spreading pattern of our model with the current rumor spreading model based on real data to prove the effectiveness of our approach. Figure 5 shows the node parameters fitting result of our proposed social network model. We implement the real-world social network database from Facebook, which includes 1899 nodes, 59835 time-stamped ties, and 20296 one-mode ties^28^. It can be observed from Fig. 5a that our proposed adapted power law distribution formula fits the out-degree centrality of nodes in real social networks well, and the out-degree centrality reflects the node’s willingness to spread, and we will define the distribution of the nodes’ willingness to spread in the model by using this fitting formula. The fit result for this dataset is $y = 0.7614 \cdot x^{-0.7617} - 7.2551$. We have categorized education levels into 7 levels based on the 2019 national education attainment data from the U.S. government website statistics, which are no education attainment, did not graduate from high school (includes students enrolled in school), graduated from high school, associate’s degree/attended college without a degree, bachelor’s degree, master’s degree/professional degree, and doctoral degree. We counted the proportions of appearances of the 7 levels. Based on our modeling setup, we attempted to find a truncated normal distribution function that makes the proportions of numbers consistent with real-world data when generating random numbers from 0 to 1. As shown in Fig. 5b, the truncated normal distribution can simulate the real education level distribution very well, and our model will generate the ability to judge false information based on this formula. The fit result for this dataset is $mean=0.4636, std=0.1818$.

**Figure 6:**
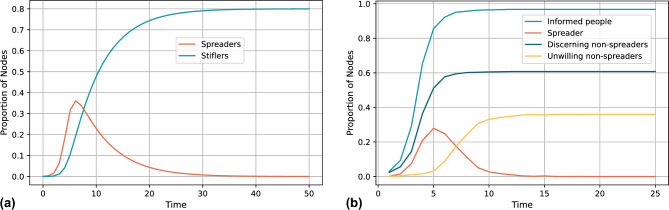
Simulation results of the false information spreading in social networks. (**a**) Existing rumor spreading model result (**b**) Our proposed model result.

LLM-generated false information can be regarded as a special kind of rumor, and in order to evaluate the ability of our model to explain the process of false information spreading in real social networks, we compare the results of our model with existing rumor spreading models^29^. The rumor model was validated based on real data and has the ability to simulate rumor spreading in the real world. We use the same BA model to generate the structure of the social network, and since the two spreading models have different definitions of the parameters, we mainly observe the pattern of the spreading process in this experiment, and the numerical results will differ due to the different model settings. We chose two spreading roles, Spreader and Stifler, in the rumor model that are similar to the metrics we evaluated. Spreader is similar to the spreader (SP) setting in our model, which indicates a person who has disseminated false information. Stifler is similar to the discerning non-spreaders (DN) setting in our model, which indicates a person who has not disseminated false information. Our model counts the total number of people who have received false information, denoted by informed people (IP), a metric that can be used to measure the overall impact of false information on the social network, and in the figure it can be observed that false information is spreading to almost all the nodes in the social network. We also refine the nodes that do not spread false information during the iteration process. Instead of DN, the unwilling non-spreaders (UN) have two sources, one is the previous spreader, who has already completed the act of spreading, and do not spread again. One is the node whose willingness to spreading is very low, even though it does not recognize false information. As time increases and the popularity of the information decreases, the node will stop spreading. As shown in Fig. 6, we show the results of false information spreading obtained from the two models, and the results show that the spreading pattern of false information is same in both models. We can observe that with time, the number of spreaders in both models increases rapidly at the beginning of the spreading and decreases rapidly to 0 after reaching the peak, while the number of people who refuse to spread the false information increases gradually until it stabilizes and then remains unchanged. The results demonstrate that our model can explain the spreading process of false information in real social networks, i.e., the dynamics of spreading from rapid growth to rapid decline, and the gradual increase in the number of rejecting spreaders until it reaches stability. The process begins with rapid spreading driven by the freshness and curiosity of the information, the number of spreaders reaches a peak and then declines rapidly as the public’s awareness of the truthfulness increases and the spreading behavior has been completed. Eventually, the number of those refusing to spread increases and stabilizes as a social consensus on the truthfulness of the information develops.

### LLM hallucination spreading modeling

**Figure 7:**
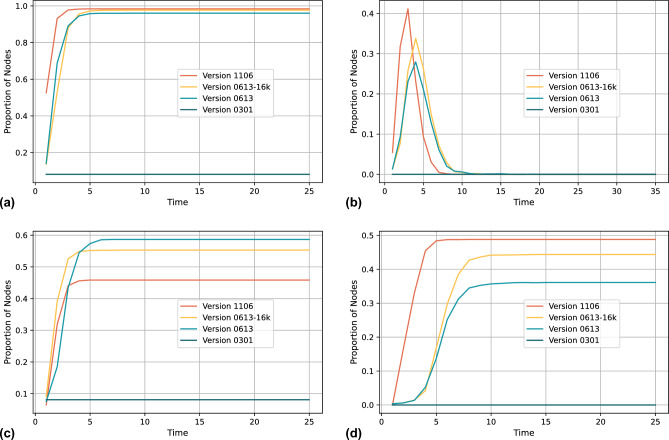
Simulation results of the LLM false information spreading for different version. (**a**) Spreading result of informed people (IP) (**b**) Spreading result of spreader (SP). (**c**) Spreading result of discerning non-spreaders (DN) (**d**) Spreading result of unwilling non-spreaders (UN).

LLM can be used for content generation in different scenarios, e.g., QA, dialogue, and summarization with different model version. Based on our previous evaluation of real LLM, the truthfulness of false information generated by LLM in different scenarios is different, in general, the truthfulness of false information generated in QA scenarios is the highest, and the nodes are the most difficult to discern, followed by summarization scenarios, and the dialogue scenarios is the easiest to discern. In addition, the new version of LLM generates false information with a higher level of truthfulness compared to the old version.

Based on our model, we first test the spreading pattern of false information generated by different versions of LLM, we choose four versions of the current mainstream LLM GPT-3.5, the version code is the release time, and we fix the generating scenario as summation. We try to simulate a social network consisting of undergraduate university students, which contains 2000 nodes. Node discernment ability is set based on their high education level, with the vast majority of nodes above 0.3, and node sharing willingness is given based on our adjusted power law distribution. We classify the node community attributes into six, which can be used to simulate student groups in different majors, such as engineering, science, literature, economics and management, medicine, and art. When the false information has the same attributes as the node, e.g., the false information is medical-related content, the medical students’ ability to recognise it will be improved, and we set the amount of change to 0.1, which indicates an improvement of one education level. When false information comes from nodes with the same attributes, for example, false information comes from students in the same major, nodes will be more likely to believe the false information, and we set the amount of change in the recognition ability to 0.1. Researchers can adjust the amount of change in the recognition ability according to the research scenario when applying our model, and we have simplified the setup because we are mainly testing the relationship between the LLM and spreading rules in this experiment.

**Figure 8:**
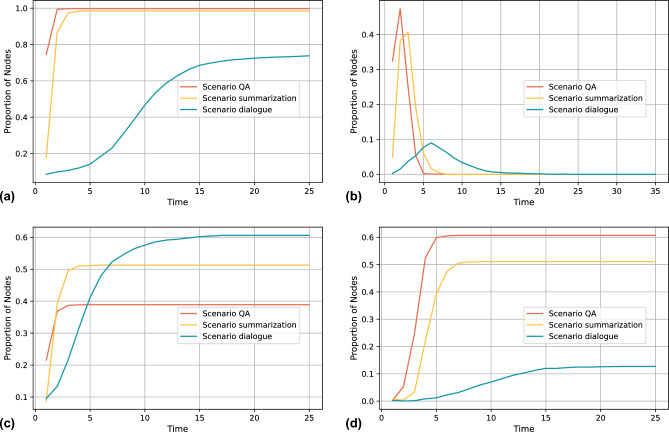
Simulation results of the LLM false information spreading for different scenario. (**a**) Spreading result of informed people (IP) (**b**) Spreading result of spreader (SP). (**c**) Spreading result of discerning non-spreaders (DN) (**d**) Spreading result of unwilling non-spreaders (UN).

Figure 7 illustrates the spreading patterns of hallucinatory false information with different versions LLMs. Each sub-graph represents a different spreading character within the network. Overall, based on the evaluation of real LLMs in our previous experiments, the newer the LLM, the more truthful the false information generated by the LLM. Figure 7a shows the proportion of nodes who are informed about the false information over time. Except for version 0301, newer versions of LLMs generate false information that is more difficult to discern, with a high degree of truthfulness. The results of these versions quickly reach a saturation point, indicating that a significant portion of the network becomes aware of the false information. Version 0301 generates false information with a truthfulness of only 0.375, due to the fact that the initial infection agent community is the same as the false information community, which has a stronger ability to recognise false information. It results in the false information generated by version 0301 affecting only the initial nodes, and there is no spreader for version 0301 as shown in Fig. 7b. The dynamics of the nodes spreading the false information over time for other versions are quickly reaching a saturation point. A sharp peak is observed for these versions, indicating a burst in spreading behaviour that quickly decays, reflecting the transient nature of active spreading among users. Figure 7c shows the proportion of nodes that are able to discern false information and therefore choose not to spread it. The curves plateau at different levels for each LLM version. Newer versions of LLM generate false information with a high truthfulness that is most difficult to recognise, and the number of nodes that can recognise it will be lower, so the newer the version, the lower the value of DN that is eventually stabilised. The DN for version 0301 is only the initial nodes, so it reaches the lowest value. Finally, Fig. 7d shows the nodes that are unwilling to spread information without discerning it. For version 0301, there is no spreading, so the value reaches zero. For other versions, since UNs come from the spreaders, false information generated by the new version of LLM is spreading with a high number of UNs in the steady state. Besides, with the help of our model, we tested the spreading of LLM false information generated in different generation scenarios with LLM GPT-3.5-1106 in the social network as shown in Fig. 8. The QA scenario and the summarization show a similar spreading pattern to the spreading of false information in previous high versions of LLM due to the high level of false information truthfulness. But it is worth noting the spreading results of false information generated in dialogue scenarios. The truthfulness of the false information generated in this scenario is 0.425, which is higher than the 0.375 of version 0301, where no spreading process occurred due to the low truthfulness of the version 0301. The false information generated in the dialogue scenario has a higher level of truthfulness, resulting in the occurrence of the spreading process, but the growth rate of its spreaders with the highest value is significantly lower than the results of the previous tests. This resulted in a very slow growth of informed people and a very low stable value of UN.

Based on our model, the researcher can set the parameters corresponding to the study scenarios and get the exact values of the spreading results. With the above experimental results, we can summarise the spreading pattern of LLMs-generated false information in social networks. The spreading pattern of false information generated by LLMs in social network is related to the situation in which the LLMs’ false content is generated. The false information generated by the new version of the more capable LLMs in a single interaction scenario such as QA and summarization is more difficult to be identified, and when the users in the social network do not have a high level of discernment ability, the false information will spread rapidly, and almost all the users in the social network will receive the false information, and the number of spreading users will increase rapidly after the start of the spreading and decrease rapidly as the spreading is finished, and these spreading users will be transformed into non-spreading users due to the fact that they have already completed the spreading behaviour and do not want to repeat the message, a portion of the nodes with high discrimination ability will identify the false information at the beginning and refuse to spread it, and the number of these nodes will increase as the spreading proceeds and stabilise at an average value at the end of the spreading. When the LLM false information is easily recognised by users due to outdated LLM version or due to dialogue scenarios, the spreading phenomenon may not occur or the rate of spreading will be significantly lower than in the previous cases and the number of spreaders will be very low and most of the nodes in the network will recognise the false information and refuse to spread it.

## Discussion

In the realm of LLMs, hallucination is becoming increasingly evident, where the system generates plausible-sounding but factually incorrect or nonsensical information. Such inaccuracies can lead to the spread of false information, especially if users trust and disseminate the AI’s responses without verification. In sensitive areas like healthcare, legal advice, or financial guidance, these hallucinations could result in harmful decisions, risking physical, legal, or financial well-being. Moreover, in educational contexts, reliance on incorrect information can impair learning and understanding. The risk is heightened when the AI’s language is authoritative or confident, as it can obscure the distinction between verified facts and generated content, potentially eroding trust in AI technology and hindering its beneficial use. Nowadays, these hallucinations occur despite models being trained on extensive datasets, as they occasionally generate responses based on patterns rather than grounded knowledge. As there are many kinds of large models and complex task scenarios, which can be set with different parameters and have multiple interactions, which will lead to uncertainty in the hallucinatory false information, in this work we try to test and evaluate the hallucinatory false information generated by the large models in different scenarios, and to apply the observed parameters to our model for quantitative spreading.

In the social network modeling approach proposed in this paper, there are limited settings for user attributes, false information attributes, and false information spreading rules, and we have considered some key underlying definitions when false information spreads and completed the modeling work. Our modeling approach allows future researchers to include more complex aspects of social networks into their analyses. To truly understand how false information from LLMs affects social networks, we need a detailed model. This model should examine how content spreads and impacts users. It’s important to accurately represent the network’s structure to grasp the intricacies of social interactions and the flow of information. The model should consider user demographics, the layout of the network, and common routes for information spread. Also, because information spread changes over time, the model must be updated regularly to remain accurate and useful. Additionally, it’s crucial to consider ethics and user privacy, following strict guidelines to protect individual rights and responsibly handle personal data. In conclusion, developing a model to quantify the impact of AI hallucinations on social networks is a multifaceted task that necessitates an interdisciplinary approach, combining technical accuracy with a nuanced understanding of human behavior and societal norms.

## Conclusion

In this paper, we propose an agent-based modeling approach for quantitatively investigating the uncertainty of hallucinatory false information spreading generated by LLMs in social networks. First, we evaluate the recognition difficulty of LLMs-generated hallucinatory false information in different situations, and we consider the effects of model version, task scenario, model temperature parameter, and model generation method on hallucinatory false information. Second, regarding the social network model, we defined network users in terms of community attributes, information recognition ability and willingness to share based on real world data. Besides, we defined false information in terms of information category, information authenticity, creation time, and popularity. Based on the above definitions and behavioral science, we set up rules for the spread of false information. Third, we tested our quantitative modeling approach based on real social network data and mainstream LLMs, evaluated the spreading of LLMs hallucinatory information in social networks under different circumstances. Finally, we discuss the current phenomenon of LLM hallucinations and how to quantitatively assess their impact on social networks.

## Data Availability

The datasets used and/or analyzed during the current study are available from the corresponding author upon reasonable request.
